# Characteristics of In2G Variant in Congenital Adrenal Hyperplasia Due to 21-Hydroxylase Deficiency

**DOI:** 10.3389/fendo.2021.788812

**Published:** 2022-01-24

**Authors:** Mirjana Kocova, Paola Concolino, Henrik Falhammar

**Affiliations:** ^1^ Medical Faculty, University “Cyril & Methodius” Skopje, Skopje, North Macedonia; ^2^ Dipartimento di Scienze di Laboratorio e Infettivologiche, Unita' Operativa Complessa (UOC) Chimica, Biochimica e Biologia Molecolare Clinica, Fondazione Policlinico Universitario Agostino Gemelli Istituto di Ricovero e Cura a Carattere Scientifico (IRCCS), Rome, Italy; ^3^ Department of Endocrinology, Karolinska University Hospital, Stockholm, Sweden; ^4^ Department of Molecular Medicine and Surgery, Karolinska Institutet, Stockholm, Sweden

**Keywords:** *CYP21A2*, c.293-13C/A>G, splicing variant, CAH, genetic counselling

## Abstract

Substantial research has been performed during the last decades on the clinical and genetic variability of congenital adrenal hyperplasia (CAH) and its most common form, 21-hydroxylase deficiency (21OHD). CAH is one of the most prevalent autosomal recessive diseases in humans, and it can be divided into classic—further subdivided into salt wasting (SW) and simple virilizing (SV)—and non-classic (NC) forms. Pathogenic variants of *CYP21A2* gene, encoding the 21-hydroxylase enzyme, have been reported with variable prevalence in different populations. NM_000500.9:c.293-13C/A>G (In2G) variant represents the most common *CYP21A2* gene changes related to the classic 21OHD form. However, the phenotype of In2G carriers is variable depending on the variant homozygous/heterozygous status and combination with other *CYP21A2* pathogenic variants. In addition, identical genotypes, harboring the homozygous In2G variant, can present with variable phenotypes including the SW and SV or rarely NC form of the disease. Here, we analyze and present the clinical aspects, genotype/phenotype correlations, and other characteristics related to the *CYP21A2* In2G variant.

## Introduction

Congenital adrenal hyperplasia (CAH) is one of the most common autosomal recessive diseases in humans with an incidence, evaluated on neonatal screening in different populations during the last decade or so, of 1:6,084 to 1:26,727 live births (1). It comprises several steroid enzyme deficiencies, among which 21-hydroxylase deficiency (21OHD) (OMIM # 201910) is by far the most common, affecting about 95%–99% of all CAH patients (1–5). In 21OHD, the hormonal disbalances consist of variable low cortisol and aldosterone levels, and compensatory high levels of 17-hydroxyprogesterone (17OHP), which converts to androgens. CAH appears in two clinical forms, classic and non-classic (NC) phenotypes (6). The classic form is further classified as salt-wasting (SW) and simple-virilizing (SV) forms. Patients affected by the SW form have SW with severe dehydration, hypoglycemia, failure to thrive, and hyperandrogenism. This can be clinically recognized early in girls due to atypical genitalia, while boys may first be identified, if not neonatally screened, when presenting with a life-threatening SW crisis within 2 weeks postnatally (2). The SV form has enough aldosterone production to avoid SW crisis and, prior to the introduction of neonatal screening, was identified due to atypical genitalia in girls and in male toddlers due to signs and symptoms of excessive androgen production, even though cases of diagnosis in adulthood occasionally happened (2, 7). The NC form is mild and can appear in numerous variants of the clinical picture, ranging from no signs in the newborn period, to mild virilization later in childhood, up to polycystic ovary syndrome or isolated hyperandrogenism and decreased fertility in adulthood (8). NC 21OHD is sometimes identified in neonatal screening (9), but most cases are identified due to symptoms and signs in adolescence or young adulthood, even though the majority probably never gets diagnosed. NC CAH shows an incidence of 1:200 to 1:1,000 (10, 11).

However, despite the traditional and generally accepted classification of CAH in different forms, due to a large variety of *CYP21A2* mutations, the phenotype can have many variants, and it is clear that CAH phenotype represents a continuum between non-classic and classic forms (12). This is important for tailoring appropriate therapy in individual patients. The 21-hydroxylase enzyme is encoded by *CYP21A2* gene, and the clinical 21OHD presentation depends upon the combination of pathogenic variants affecting this locus (13). Both *CYP21A2* gene and *CYP21A1P* pseudogene are located on the short arm of chromosome 6 (6p21.3), in the human leukocyte antigen (HLA) class III region of the major histocompatibility (MHC) locus. These genes contain ten exons spaced over 3.4 kb with a sequence homology reaching 98% (12, 14, 15). Intergenic recombination events represent more than 95% of pathogenic variants causing 21OHD. Approximately 75% of the deleterious variants are transferred by small conversions from the pseudogene during meiosis (16, 17). Of the cases of 21OHD, 20%–25% are due to gene deletions, gene duplications, and deletions involving *CYP21A2* and other contiguous genes (18). Finally, *CYP21A2* pathogenic variants that are not apparently gene conversions account for 5%–10% of CAH alleles in most populations (16, 17).

To date, more than 230 *CYP21A2* pathogenic variants have been identified (18). Most patients are compound heterozygous; and in this case, the phenotype correlates with the variant that predicts the higher residual enzyme activity (19). Based on the residual activity of the mutant enzyme, *CYP21A2* variants are classified into specific groups (null, A, B, and C) (20, 21). While variants of the null group show 0% enzyme activity during *in vitro* assay, group A variants preserve a minimal (<1%) residual activity. These two groups are both associated with the SW form of 21OHD. Differently, group B (1%–5% enzyme activity) and group C (20%–50% enzyme activity) variants are related to the SV and NC forms, respectively (12, 22–25). Although there is good agreement between clinical phenotype and patient genotype, it is well-known that some exceptions exist (18–29).

The prevalence of 21OHD is highly variable among populations; some countries show higher (China and India) (19, 20) or lower prevalence (Japan and New Zealand) (3, 21–27). However, in most of the analyzed studies, the In2G variant is found to be, with rare exceptions (30–50), among the most common *CYP21A2* pathogenic variants. It is usually related to the SW 21OHD; however, patients with a non-correspondent phenotype have been widely reported. In this review, we collected the most relevant evidence showing the phenotypic variability of the In2G variant.

## *CYP21A2* In2G Variant

**Figure 1:**
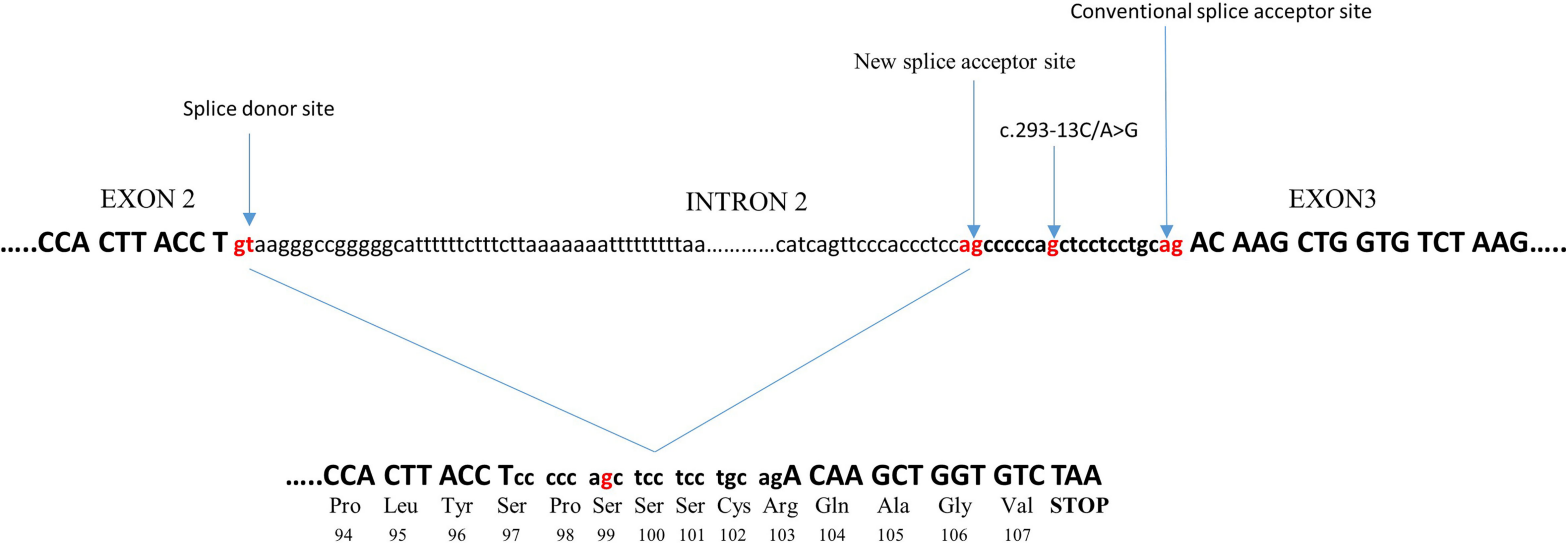
Effect of the c.293-13C/A>G variant on splicing. The c.293-13C/A>G variant, at a −13 position before the end of intron 2, creates an additional splice acceptor site, causing aberrant splicing of intron 2 with retention of 19 intronic nucleotides. This causes a frameshift in the following third exon to generate a stop codon after 107 amino acids, resulting in the production of a truncated protein.

To date, 18 intronic splicing variants, representing 7.7% of all disease-causing variants, have been reported in *CYP21A2* gene (18, 51). *In silico* analysis or functional studies showed that all these variants are associated with the severe form of 21OHD due to the changed reading frame of the gene producing a non-functional enzyme (21, 52, 53). Generally, an intronic splicing variant causes the disruption of the acceptor/donor site, inducing activation of an intronic cryptic acceptor/donor site, retention of a whole intron or part of it, and exon skipping (51). Regarding intron 2 of *CYP21A2* gene, five pathogenic splicing variants have been reported (51). Two of these, c.292+1G>A and c.293-2A>G, cause the SW phenotype to disrupt the donor and acceptor splicing sites, respectively (54, 55). Differently, the c.292+5G>A and c.293-7C>G variants were described, in SW patients, as reducing the consensus value for the intron 2 splice donor and acceptor sites, respectively (52, 56).

c.293-13C/A>G (In2G) is the most common splicing variant in *CYP21A2* gene. It is usually transferred by microconversion from *CYP21A2* pseudogene. At the −13 position, before the end of intron 2, the wild-type nucleotide is A or C. Substitution to G creates an additional splice acceptor site, causing aberrant splicing of intron 2 with retention of 19 intronic nucleotides. This results in a shift in the translational reading frame (21) (**Figure 1**). The In2G variant is typically related to the SW form of CAH. It is by far the most common CAH mutation in the majority of countries and ethnicities, predominantly Europeans followed by Middle Easterners and Hispanics (27), although there are exceptions (**Table 1**). In different studies, the prevalence of the In2G variant was between 20.6% and 30.3% (25, 27, 61, 63). In the homozygous form, it is more common in those with European and Middle Eastern ancestries than in Hispanic Americans, Asians, or East Indians (27). Some authors even refer up to 60.4% prevalence of this variant in certain ethnicities (32) (**Table 1**).

**Table 1:** Prevalence of In2G variant in different countries.

Country	In2G (%)	No. of alleles	Reference
Argentina	35.2	866	(25)
Austria+Germany	29.2	1,320	(57)
Brazil	21.1	856	(20)
Chile	5.3	38	(35)
China	35	460	(58)
Czech Republic	45.4	174	(48)
Croatia	34.9	186	(49)
Cuba	24.5	110	(59)
Denmark	33.8	136	(36)
France	10.9	247	(37)
Finland	9.6	156	(44)
Greece	29.3	222	(50)
Italy	21.1	114	(38)
India	48	124	(47)
Iran	14.7	88	(39)
Japan	26.5	136	(43)
Turkey	22	112	(45)
Mexico	47.9	94	(60)
Netherlands	28.1	370	(61)
North Macedonia	60.4	48	(32)
Rome population	95	20	(62)
Romania	43.9	86	(40)
Serbia	18.5	122	(33)
Spain	17.5	354	(41)
Sweden	26.6	400	(63)
United Kingdom	30.3	284	(46)
United States	22.9	3,005	(27)
Mid-Europe	31.2	864	(42)

In some populations, the In2G variant could be considered a founder variant. It is a unique finding in some enclosed populations such as Alaskan and one of the Roma populations from the Balkan region, although the number of explored individuals was rather small (62). Finally, in other populations, such as the Spanish population, the In2G variant appears to be related to recent conversion events (64).

### Genotype/Phenotype Correlation in In2G Patients

There is a reasonable genotype/phenotype correlation in 21OHD despite disease-causing variant variability (3, 28–30). As mentioned previously, the phenotype is almost uniformly dependent on the milder variant in the genotype. However, some variants may occasionally confer an unexpected phenotype (27, 63, 65).

In the homozygous status or *in trans* with another null variant, the In2G variant usually causes severe SW CAH. When combined with a moderate or mild variant, it normally confers SV or NC CAH. However, a specific characteristic of the In2G variant is that the clinical picture might be less severe even in the homozygous form when it can present as an SV or NC phenotype (22, 24, 31, 32).

During the 1990s, the first evidences about the phenotypic heterogeneity related to the In2G variant were reported (66, 67). Witchel et al. compared the clinical and molecular findings in 38 individuals from 21 families. All patients carried two deleterious variants *in trans*, with the In2G present on at least 1 allele. A comparison of the phenotypic features with the molecular genotypes showed phenotypic heterogeneity extending from classic SW 21OHD to be asymptomatic (28). The authors hypothesized that other sequence variations influenced the competitive splicing signals at the intron 2/exon 3 junctions. However, experimental testing did not support this hypothesis, and the molecular basis of the phenotypic heterogeneity associated with the In2G variant remained to be elucidated (28). A few years later, Schulze et al. suggested that the putative asymptomatic In2G homozygous individuals were incorrectly typed due to the dropout of one allele during PCR amplification (68). Effectively, in the 1990s, it was too challenging to accurately genotype *CYP21A2*, which still presents as one of the most difficult and error-prone genes, even today. In fact, many old *CYP21A2* genotyping results have been found to be incomplete or inaccurate by using up-to-date methodologies. For this reason, it could be necessary to re-evaluate the accuracy of some of the old literature. However, even with the use of more sophisticated techniques for genotyping, patients with the In2G variant and a non-correspondent phenotype have been reported. In a large study by New et al., out of 155 homozygous In2G patients, 143 (92.3%) had the SW form, 11 (7%) had the SV form, and 1 (0.6%) had the NC form (27). Even when the In2G variant was detected *in trans* with another severe mutation, such as p.(Gln319Ter), still 12% of patients (3/25) presented as SV (27). Finally, the genotype In2G/p.(Val282Leu) was related to the NC phenotype in 96.4% of patients, and only 4 (3.6%) subjects presented a severe phenotype (27). In a recent study by Riedl et al., of 62 In2G homozygous patients, 53 (85.5%) had the SW form, whereas 9 (14.5%) had the SV form (57). DumiK et al. described a comprehensive *CYP21A2* mutation analysis in a large cohort of 93 Croatian patients with classic 21OHD (49). The most frequently detected mutation in this population was the In2G variant (34.9%) (**Table 1**). The concordance between observed and predicted clinical phenotype in Group A (In2G variant) patients was 85% (49). In particular, the authors described two families with genotype–phenotype discordance (49). In the first family, three sisters carried the In2G/In2G genotype. Two of them displayed an SW phenotype and were on hydrocortisone and 9-alpha-fludrohydrocortisone therapy. In contrast, the middle sisters had ambiguous genitalia, high levels of 17OHP and androgens, but repeat measurements of electrolytes, aldosterone, and plasma renin activity (PRA) were within the normal range, excluding the SW phenotype. In the second family, two siblings showed the In2G/p.(Arg358Trp) genotype. The brother was diagnosed with SV 21OHD at 3 years of age due to precocious pseudopuberty, high levels of 17OHP and androgens, and a normal level of aldosterone and PRA. His sister was diagnosed with SW CAH at birth, as she showed ambiguous genitalia, low levels of sodium and aldosterone, and high levels of potassium and PRA. In this case, hydrocortisone and 9-alpha-fludrohydrocortisone were introduced 10 days after birth (49). Also, in Argentinean CAH patients, the In2G variant was reported as the most prevalent mutation (35.2%) (**Table 1**), and while 83.8% of patients in group A (In2G variant) presented with the SW form, 16.2% showed the SV form of the disease (25). In this regard, also these authors described two siblings with the same genotype (In2G/In2G) but a different phenotype (25). Similar data from Brazilian, Hellenic, and Chinese CAH populations were provided by Carvalho et al., Dracopoulou-Vabouli et al., and Wang et al., respectively (**Table 1**) (20, 50, 58).

The In2G variant, in homozygous or *in trans* with a severe *CYP21A2* mutation, was also related to the NC form of 21OHD. Bidet et al. analyzed the molecular spectrum of *CYP21A2* gene in a large cohort of French NC CAH patients (37). The In2G variant was present on 10.9% of all chromosomes (**Table 1**), making it the second most frequent mutation in this study. Interestingly, the authors described a mild clinical and biological phenotype, related to the NC form of 21OHD, in a patient homozygous for the In2G variant.

Finally, a peculiar case was reported by Kohn et al. (55). These authors described two affected boys, both carrying the In2G/In2G genotype, who thrived in early infancy but suffered SW crises unusually late in infancy, at 3.5 and 5.5 months. At the onset of symptoms, the children showed hyponatremia, hyperkalemia, dehydration, and acidosis; serum aldosterone was low in spite of markedly elevated PRA. Baseline 17OHP levels were only moderately elevated; however, stimulated levels were consistent with the classic form of 21OHD. The authors speculated that the In2G variant could sometimes be associated with the delayed phenotypic expression of SW CAH and that the variable splicing may modify the clinical manifestations of the disease (55).

### Outcomes of In2G Patients

Although all variants causing SW 21OHD induce similar clinical picture, require similar therapy, and produce similar outcomes, some of the specificities of the In2G variant are being confirmed in a number of larger studies. Here, we will mention some of them.

#### Fertility

Fertility is significantly decreased in all genetic forms of SW CAH in women due to high levels of androgens, problems after genital surgery, decreased sex drive, social adaptation issues such as not having a partner, or non-willingness to bear children (69). Only approximately 25% of women with CAH conceive a child compared with 45% of matched controls (70, 71). They give birth mostly by cesarean section (72%) and are prone to gestational diabetes. Elevated androgen concentrations impair the ability of progesterone to lower the activity of gonadotrophin-releasing hormone/luteinizing hormone (GnRH/LH) pulse generator, causing increased frequency of pulse amplitude of LH over follicle-stimulating hormone (FSH) production and also disrupting endometrial thickening, making the cervical mucus thicker, disrupting ovulation, and impairing embryo implantation, which all lead to impaired fertilization (69). Psychosexual factors also have a role. Women with CAH frequently present with masculine behavior, and approximately one-third do not have sexual interest and fantasies (72). Moreover, homosexuality is more common in women with CAH, and there is a direct relationship between the severity of the genotype and non-heterosexuality (73, 74). For example, in women with the null genotype, 50% had a non-heterosexual orientation; in the In2G genotype, 30%; and in matched controls, only 2% (74). Women with null and In2G genotypes were less often married and had fewer children than women with milder genotypes (75). However, fertility in women with the In2G genotype was better compared with the null genotype (71). Pregnancy in women with SW CAH was normal, and the offspring had a normal weight and development (76). The better fertility in females with In2G might have to do with the dose-dependent effects of prenatal androgens on the development of higher brain functions (74). Females with the null genotype scored lower on sexual function and satisfaction with their sexual life as well as had more genital surgical complications, compared with the In2G genotype (77).

Fertility is also compromised in males with CAH, mostly due to testicular adrenal rest tumor (TART) or sometimes hypogonadotropic hypogonadism (78). However, the remaining testicular tissue is larger, and the amount and quality of semen are better in patients with the In2G variant, although not reaching statistical difference (79), with male SW CAH having an increased number of adopted children (80). Nevertheless, the number of males with at least one biological child was equally low in both the null and In2G genotype groups (80).

#### Psychiatric Disorders

Research in animal models has demonstrated that sex differences in brain and behavior are induced by steroid hormones during specific, hormone-sensitive developmental periods (81). Steroid hormones permanently organize the brain for gender, including the pattern of sexuality, cognition, temperament, and specific interests according to sex, although these features can be modified by environmental and social factors (82). It has been demonstrated that typical male neural and behavioral characteristics develop under the influence of testosterone during perinatal development (81). The fetal hyperandrogenemia in females with CAH leads to male brain organization and subsequently to masculinized behavior and cognitive function (72, 83). Significant psychologic issues originate from these brain compositions in female patients with CAH, and they are dependent on the amount of prenatal and perinatal androgen levels. On the other hand, the disturbed hypothalamic–pituitary–adrenal (HPA) axis in patients with CAH may result in a hypersensitive stress system, making them vulnerable to addiction (84). Thus, three major psychiatric disturbances are present in female patients with CAH: high risk for psychiatric disorders, substance misuse disorders, and stress-adjustment disorders (85). Some authors find psychologic and psychiatric disturbances most expressed in patients with the null genotype (86). Having in mind the symbolic level of the 21-hydroxylase enzyme produced in some patients with the In2G genotype due to alternative splicing, it is expected that they will be less prone to psychologic or psychiatric disorders. In the study of Mueller et al. on a large sample of female patients with CAH, 44.4% met the criteria for at least one psychiatric diagnosis (87). Similar findings were reported in 221 adult females with CAH from six European countries (88). In the study of Engberg et al., females with CAH had high levels of psychiatric disorders as compared with matched controls, and interestingly, patients with the In2G mutation genotype were slightly more frequently affected than the null genotype (85). On the other hand, substance misuse, alcohol, drugs, and attention-deficit hyperactivity disorder were more frequent in patients with the null genotype. In contrast, in males with the In2G genotype, psychiatric disorders, personality disorders, and alcohol misuse were increased as compared with the null genotype (89). Similar findings were reported by Daae et al. (90). The reason for such discrepancy remains elusive. Interestingly, the parents of children with severe genotypes including null and In2G, i.e., obligate *CYP21A2* variant carriers, are at much lower risk of being diagnosed with psychiatric disorders (91).

#### Cardiovascular and Metabolic Disorders and Bone Health

As far as the metabolic outcomes and complications such as obesity, cardiovascular complications, and bone fragility, they are mostly associated with the therapy; therefore, delineating the influence of genotype is very complicated due to different treatment regimens and length of therapy. However, the risk is generally increased as compared with controls (92–95). In one large epidemiological study, only obesity and venous thromboembolism were significantly more common in patients with In2G than in controls, while patients with null variants had more cardiometabolic risk (96).

## Discussion

The In2G variant is frequent in patients with 21OHD. It normally causes severe disease; however, the clinical presentation can vary from the SW form, through the SV form and rarely the NC form. Thus, there is a difference in the severity of 21OHD within group A. The mechanism underlying the variation in the clinical phenotype of the In2G variant was widely discussed. The most accredited hypothesis is that a small number of transcripts avoid aberrant splicing, providing a small amount of the 21-hydroxylase enzyme, which is sufficient for a milder clinical presentation of the disease. For this reason, in some patients, the phenotype appears as SV or even NC. *In vitro* experiments showed that the *CYP21A2* intron 2 c.292+1G>A variant produces two different transcripts: the type I fragment lacked the entire introns 1 and 2 and exon 2, whereas the type II, representing approximately one-third of the mRNAs produced and generated by the use of a cryptic splice acceptor site downstream from exon 3, had a deletion of intron 1, entire exon 2, and part of intron 2 (97). These results supported the evidence that splicing is not a homogenous mechanism and that a single splicing variant can generate alternative transcripts, which might explain some unusual phenotypes (97, 98). In addition, the potential influence of extra-adrenal 21-hydroxylation on the CAH patient’s phenotype might be an additional cause to consider. In fact, it was demonstrated that hepatic CYP2C19 and CYP3A4 have the ability to 21-hydroxylate progesterone and thus may modulate mineralocorticoid deficiency (99).

In conclusion, although a good genotype–phenotype correlation exists in 21OHD, a disparity in phenotypic appearance is present in a portion of patients carrying the In2G/In2G or In2G/null genotypes. This evidence represents the most challenging issue in prenatal diagnosis and familiar counselling since the predictive value for different phenotypes can be uncertain.

## Author Contributions

MK drafted the manuscript and participated in writing and editing. PC participated in writing and editing. HF participated in writing and editing. All authors contributed to the article and approved the submitted version.

## Funding

This study was supported by Magnus Bergvall Foundation.

## Conflict of Interest

The authors declare that the research was conducted in the absence of any commercial or financial relationships that could be construed as a potential conflict of interest.

## Publisher’s Note

All claims expressed in this article are solely those of the authors and do not necessarily represent those of their affiliated organizations, or those of the publisher, the editors and the reviewers. Any product that may be evaluated in this article, or claim that may be made by its manufacturer, is not guaranteed or endorsed by the publisher.
